# Using Neutrons to Elucidate the Catalytic Shift from Superoxide Dismutase to Peroxidase Activity in Fe-Substituted Human MnSOD

**DOI:** 10.1063/4.0000905

**Published:** 2025-10-27

**Authors:** Miles Graham, Gloria E. O. Borgstahl

**Affiliations:** University of Nebraska Medical Center, Omaha, NE, USA

## Abstract

Human manganese superoxide dismutase (MnSOD2) is a metallo-oxidoreductase localized to the mitochondrial matrix. Its canonical function is as an antioxidant, neutralizing superoxide radicals generated during the electron transport chain (ETC). By converting superoxide into hydrogen peroxide and molecular oxygen, MnSOD2 safeguards sensitive metabolic enzymes from oxidative damage and facilitates mitochondrial redox signaling via membrane-diffusible hydrogen peroxide. At the core of MnSOD2’s activity is a proton-coupled electron transfer (PCET) mechanism driven by the cyclical oxidation and reduction of the active-site Mn.

The clinical relevance of MnSOD2 is contradictory: downregulation promotes tumorigenesis, while upregulation promotes increased malignancy and metastatic activity in established tumors. Recent investigations into the mechanism underpinning this dichotomy have suggested the erroneous metal incorporation could be to blame. When MnSOD2 is overexpressed, enzyme levels outstrip intracellular Mn reserves and it begins incorporating Fe in lieu of Mn, becoming FeSOD2. While FeSOD2 has long been known to be catalytically dead towards superoxide, recent studies have uncovered it gains prooxidant peroxidase activity instead. Clinically, FeSOD2 has been observed acting as a stemness- promoting histone demethylase in breast cancer.

Despite its importance, little headway has been made towards understanding the atomic mechanism of this catalytic shift from SOD to peroxidase. One of the major challenges in elucidating the mechanism is the inherent difficulty of studying metallo-oxidoreductases and PCETs using traditional structural techniques like X-ray crystallography. X-rays often reduce sensitive metal centers, preventing the uncovering of the mechanically critical oxidized intermediates. Furthermore, protons are insensitive to X-ray diffraction, making the technique unsuited for identifying labile protons in PCET reactions. We aim to overcome these issues using neutron crystallography. Neutrons are non-ionizing, making them safe to use on oxidized metal centers as they won’t cause radiation damage or photoreduction. Hydrogens also have a similar scattering cross-section to carbon, making them readily apparent in nuclear density maps. This ability to directly image proton locations makes neutron diffraction ideal for studying PCET mechanisms.

Our lab has previously used macromolecular neutron diffraction studies to elucidate the PCETs underpinning the mechanism of MnSOD2, and we now seek to use a similar methodology to uncover how the atomic mechanism changes upon Fe incorporation. In preparation for our neutron studies, we have solved X-ray diffraction structures of oxidized, reduced, and H¬¬2O2-bound FeSOD2 to provide a foundation for interpreting future neutron crystallographic data. Additionally, we used X-ray absorption spectroscopy (XAS) to determine the molecular orbitals, coordination state, oxidation number, and interatomic distances of the metal center of oxidized, reduced, and substrate-bound FeSOD2. Along with directly informing the electronic state of the metal in catalytically relevant states, this data will provide refinement constraints for our neutron diffraction structures.

Our X-ray crystallography data shows that we can successfully manipulate the redox state of FeSOD2 crystals, and that the substrate binds to the Fe center. XAS data shows that the metal center can oscillate between 2+ and 3+ oxidation states. These results are encouraging for our future neutron crystallography and indicate a high likelihood of success. By elucidating this mechanism, we hope to improve our understanding of redox biology and present a potential therapeutic target for SOD2-enriched malignancies.

